# Fluoroscopic characterization of pectus excavatum in dogs: a cross-sectional study

**DOI:** 10.1093/jvimsj/aalag171

**Published:** 2026-08-24

**Authors:** Minjoo Kwon, Nayeong Park, Youngah Choi, Jihye Choi, Junghee Yoon

**Affiliations:** College of Veterinary Medicine and the Research Institute for Veterinary Science, Seoul National University, Seoul, South Korea; College of Veterinary Medicine and the Research Institute for Veterinary Science, Seoul National University, Seoul, South Korea; College of Veterinary Medicine and the Research Institute for Veterinary Science, Seoul National University, Seoul, South Korea; College of Veterinary Medicine and the Research Institute for Veterinary Science, Seoul National University, Seoul, South Korea; College of Veterinary Medicine and the Research Institute for Veterinary Science, Seoul National University, Seoul, South Korea

**Keywords:** canine, bronchial collapse, tracheal collapse, lung herniation, fluoroscopy

## Abstract

**Background:**

The dynamic respiratory abnormalities in dogs with pectus excavatum are incompletely characterized.

**Hypothesis/Objectives:**

To characterize fluoroscopic findings in dogs with pectus excavatum and evaluate their associations with clinical severity.

**Animals:**

Fifty-three client-owned dogs with pectus excavatum.

**Methods:**

Cross-sectional study. Dogs were classified into low-severity or high-severity groups based on respiratory signs and treatment. Fluoroscopic examinations were evaluated for pectus excavatum-intrinsic findings and concurrent respiratory abnormalities. Associations with clinical severity were assessed using univariable and multivariable analyses.

**Results:**

Multiple fluoroscopic findings of pectus excavatum were characterized. Paradoxical sternal movement was independently associated with clinical severity (odds ratio [OR] 3.71, 95% CI, 1.03-13.4; *P* = .045). Thoracic height reduction ratio was significantly higher in the high-severity group (0.140 [IQR, 0.100-0.228]) compared with the low-severity group (0.097 [IQR, 0.064-0.133]; *P* = .04). Mainstem bronchial collapse was present in all dogs (53/53, 100%) and cervical lung lobe herniation in 50/53 (94%; 95% CI, 84%-99%). Most other fluoroscopic variables showed no significant associations with clinical severity.

**Conclusions and clinical importance:**

Paradoxical sternal movement, particularly with greater thoracic height reduction, may serve as a supportive indicator of clinical severity. The high prevalence of bronchial collapse and cervical lung lobe herniation suggests an association between pectus excavatum and these airway abnormalities in dogs.

## Introduction

Pectus excavatum (PE) is a common sternal deformity in dogs, characterized by dorsal deviation of the sternebrae and dorsoventral narrowing of the thorax^1^. Although several embryologic theories exist—including costal cartilage overgrowth, sternal growth arrest, and abnormal diaphragmatic traction—no single mechanism fully explains the deformity in dogs.^2^^,^^3^ In humans, PE is associated with relevant cardiopulmonary dysfunction through pulmonary compression causing restrictive ventilation and right ventricular compression leading to reduced cardiac output.^4^^,^^5^ Surgical decision making in humans incorporates multiple criteria including clinical signs, progression history, paradoxical chest wall motion, Haller index in computed tomography greater than 3.25, and evidence of cardiac or pulmonary compression.^6^ The Haller index is calculated as the maximal internal transverse diameter of the chest divided by the minimal anteroposterior diameter at the same level.^7^ However, the phase of the respiratory cycle markedly affects morphological measurements, with inspiration yielding Haller index values significantly lower than expiration, emphasizing the importance of dynamic assessment.^8^^,^^9^

Despite the well-established clinical relevance of PE in human medicine, its clinical importance in veterinary medicine remains poorly understood. Few studies have investigated the specific factors that determine clinical severity in PE in dogs, including paradoxical chest wall motion and deformity characteristics. Unlike human studies, the association between PE and concurrent airway disorders such as tracheal collapse (TC) and mainstem bronchial collapse (BC) has not been clearly established in dogs. Additionally, although some breed predispositions and morphological correlations are reported,^10^^,^^11^ comprehensive fluoroscopic evaluation of respiratory dynamics in dogs with PE remains limited.

The aims of this study were to (1) comprehensively characterize fluoroscopic findings in dogs with PE, including both PE-intrinsic findings and concurrent respiratory abnormalities, and to (2) determine which, if any, fluoroscopic findings were associated with clinical severity.

## Materials and methods

### Animals

This cross-sectional study included client-owned dogs presented to the Seoul National University Veterinary Medical Teaching Hospital between January 2019 and April 2025 that were diagnosed with PE on thoracic radiograph. Cases were included when dynamic fluoroscopy captured the respiratory cycle with the sternum within the field of view (FOV) and manual tracheal stimulation was performed to assess airway collapse, and cases without manual tracheal stimulation or with inadequate sternal coverage on fluoroscopy were excluded. When multiple examinations were available, the first fluoroscopic examination was selected.

### Fluoroscopic examination

Fluoroscopic examinations were obtained with a C-arm (SPINEL 3G; GEMSS Healthcare Corporation, Paju, Republic of Korea) with dogs positioned in right lateral recumbency and in a ventrodorsal (VD) position. For each dog, fluoroscopic cine loops were recorded during quiet tidal breathing and during a coughing maneuver. The coughing phase was induced by manual tracheal stimulation to enhance visualization of dynamic airway collapse. All images were archived in the Picture Archiving and Communication System (PACS) in Digital Imaging and Communications in Medicine (DICOM) format.

### Fluoroscopic image analysis

All fluoroscopy was reviewed on a PACS workstation using electronic calipers. Three observers (with 1-2 years of training in veterinary radiology) evaluated each study independently. The observers were blinded to each other’s results and clinical histories. Right lateral views were used for measurements of thoracic height and diaphragmatic motion; VD views (forelimbs positioned caudally) were used for assessment of cervical lung lobe herniation (CLLH) and cardiac displacement.

Evaluation criteria were categorized into 2 groups: (1) PE-intrinsic findings (the thoracic height reduction ratio [THRR], the location of the sternal deformity, and cardiac displacement) and (2) concurrent respiratory findings (paradoxical sternal movement, the diaphragmatic excursion ratio, the regional grade of TC, the presence of BC, CLLH, and tracheal kinking). This distinction was made because respiratory findings might occur in dogs with or without PE and might be influenced by concurrent respiratory diseases or breed-specific factors.

**Figure 1:**
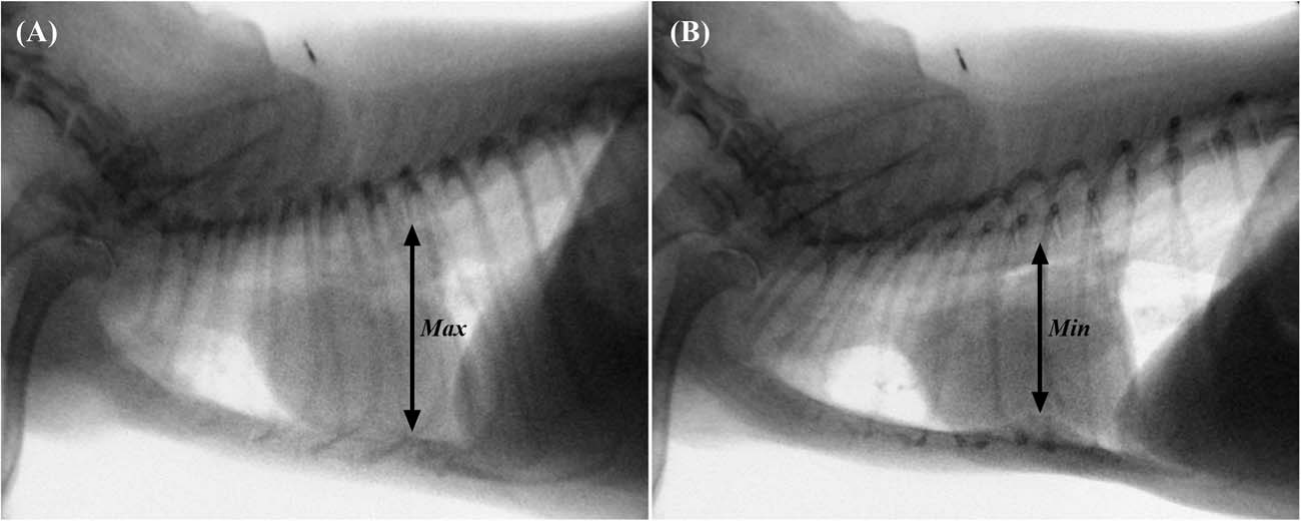
Measurement of THRR using 2 frames at the extremes of sternal excursion during respiration. (A) End-expiratory frame showing sternum at its most ventral position. The double-headed arrow indicates thoracic height measured as the shortest distance from the most severely deformed point of the sternum to the ventral cortex of the thoracic vertebral body at the same level. (B) End-inspiratory frame showing sternum at its most dorsal position with thoracic height measured using the same method. THRR was calculated as (Height_max − Height_min)/Height_max. Abbreviation: THRR, thoracic height reduction ratio.

Thoracic height reduction ratio, which quantifies the dynamic change in sternal-vertebral distance during respiration similar to respiratory phase-dependent Haller index measurements in humans,^8^^,^^12^ was measured in right lateral recumbency by selecting 2 frames at the extremes of sternal excursion (sternum most dorsal and most ventral). On each frame, thoracic height was the shortest distance from the most severely deformed point of the sternum to the ventral cortex of the thoracic vertebral body at the same level (Figure 1). Thoracic height reduction ratio was calculated as (Height_max − Height_min)/Height_max.

**Figure 2:**
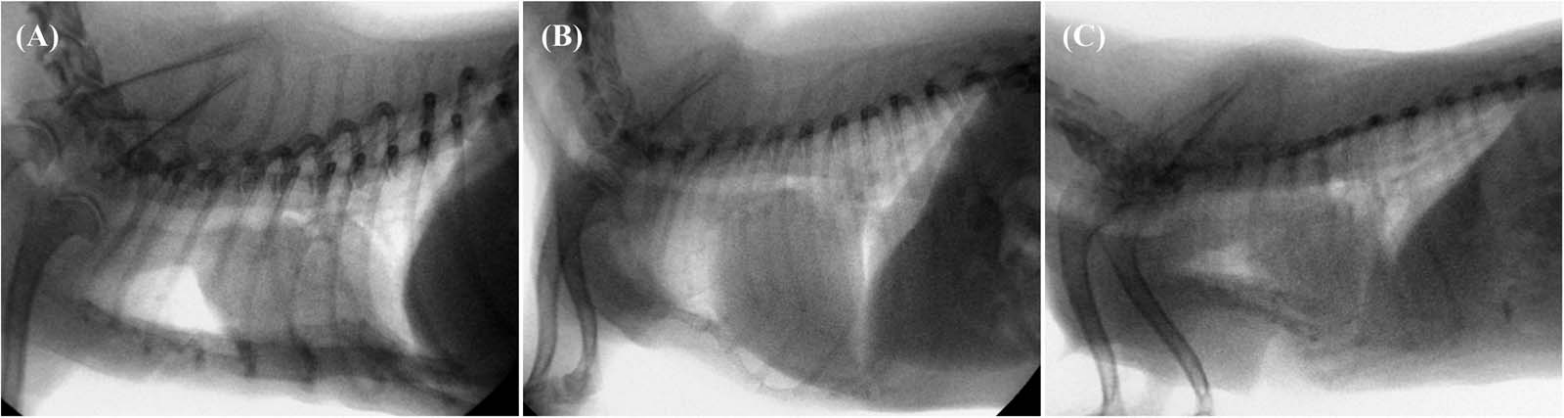
Classification of pectus excavatum based on location of sternal deformity. (A) Typical deformity with caudal sternebrae primarily affected. (B) Atypical deformity with cranial sternebrae (manubrium to fourth sternebra) primarily affected. (C) Atypical deformity with multiple sternebrae producing a generalized concave sternum.

Location of the sternal deformity was classified as typical when caudal sternebrae were primarily affected. It was classified as atypical when the maximal displacement was cranial (manubrium–fourth sternebra) or when the deformity extended across multiple sternebrae producing a generalized concave sternum (Figure 2).^11^

Cardiac displacement was assessed on VD end-inspiratory fluoroscopic view and considered present when the cardiac apex deviated laterally (typically leftward) from the vertebral midline. If the heart was outside the FOV, the VD thoracic radiograph acquired the same day at maximal inspiration was used for this assessment.

Paradoxical sternal movement was recorded when the sternum moved inward (dorsally) on inspiration and outward (ventrally) on expiration on the right lateral view (Video SS1, see Supplementary Data).

**Figure 3:**
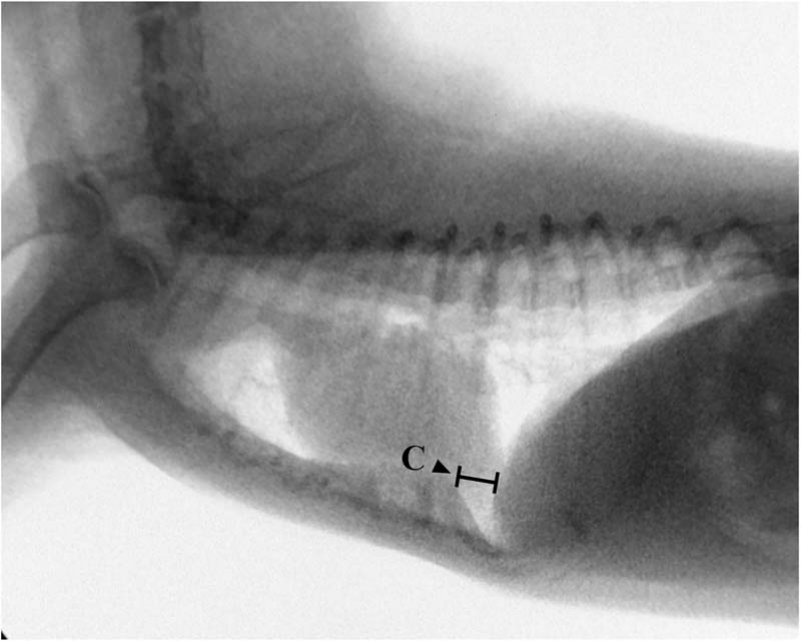
Measurement of DER on fluoroscopy. The double-headed arrow indicates excursion, measured as the shortest straight-line distance between the diaphragmatic cupula (C) at end-expiration and end-inspiration. DER was calculated as excursion distance divided by T8 vertebral body length from the same image. Abbreviations: DER, diaphragmatic excursion ratio; T8, eighth thoracic vertebra.

Diaphragmatic excursion ratio (DER) was measured in right lateral recumbency. Excursion was defined as the shortest straight-line distance between the diaphragmatic cupula at end-expiration and at end-inspiration. Diaphragmatic excursion ratio was calculated as excursion divided by the T8 vertebral body length from the same study (Figure 3). Based on prior reports in normal dogs,^13^ DER was categorized into 3 groups: reduced excursion (DER < 0.47), normal excursion (DER 0.47-0.82), and increased excursion (DER > 0.82).

Tracheal collapse was graded in the cervical, thoracic inlet, intrathoracic, and carinal regions by measuring the maximum and minimum intraluminal diameters of the trachea during dynamic fluoroscopic examination. Following cough induction through gentle tracheal palpation or spontaneous coughing, TC was assessed throughout the respiratory cycle, including both inspiratory and expiratory phases, to capture collapse occurring at different phases in different regions. Diameter reductions of 0%-25% were classified as grade 1, 25%-50% as grade 2, 50%-75% as grade 3, and 75%-100% as grade 4.^14^ For statistical analysis, TC grades were categorized as mild (grades 1 and 2), moderate (grade 3), and severe (grade 4).

Mainstem bronchial collapse was recorded when bronchial diameter decreased by ≥ 60% throughout the respiratory cycle.^15^ Cervical lung lobe herniation was recorded when the apical level of the extruded lung lobe was cranial to the seventh cervical vertebra (Video SS2, see Supplementary Data).^16^ Tracheal kinking was recorded when the trachea lost its linear course and assumed a curved or angulated path (Video SS3, see Supplementary Data).^17^

For ordinal/nominal criteria (TC grades, BC, CLLH, tracheal kinking, paradoxical sternal movement, deformity location, cardiac displacement, and diaphragmatic excursion group), the final determination was the majority decision of the 3 observers (≥2 in agreement). Cases with no majority, or with TC grades differing by 2 or more levels, were reviewed jointly and a consensus determination was recorded. For continuous measures (THRR), the 3 observer measurements were averaged to obtain the final value.

### Medical records

For each dog, signalment, respiratory clinical signs (such as presence of chronic cough, cyanosis or syncope, and respiratory distress), whether respiratory medication including bronchodilators, anti-inflammatory drugs, or antitussives was prescribed were recorded. Each dog was classified into 2 clinical severity groups based on the treatment medication prescribed: low-severity group (mild or no signs of respiratory disease, no respiratory medication prescribed) and high-severity group (severe signs of respiratory disease, requiring respiratory medication).

Clinical indications for fluoroscopic examination were recorded from medical records. Dogs were categorized as having either respiratory-related indications (evaluation of respiratory signs for suspected TC or bronchial collapse) or non-respiratory indications (screening before anesthesia for surgical procedures in the absence of respiratory complaints).

### Statistical analysis

All statistical analyses were performed using IBM SPSS software version 25.0 (IBM Corp., Armonk, NY, USA). Fisher’s exact or chi-square tests were used for categorical variables, and the Kruskal–Wallis test was used for continuous variables, with Mann–Whitney *U* tests for pairwise comparisons as needed. Statistical significance was considered at a *P* value of < .05.

Interobserver reliability was assessed using the original independent ratings from all 3 observers before any consensus discussion. The consensus process described above was used only for determining final decisions, not for reliability analysis. Intraclass correlation coefficients (ICCs) were calculated for continuous variables (THRR and DER). Weighted kappa coefficients with quadratic weights were calculated for ordinal variables (TC grades), and Fleiss’ kappa was calculated for binary variables (cardiac displacement). Reliability was not assessed for sternal deformity location, BC, CLLH, tracheal kinking, and paradoxical sternal movement, as all 3 observers achieved complete agreement for these variables in all cases. Intraclass correlation coefficient values of < 0.40, 0.41-0.60, 0.61-0.80, and > 0.80 were interpreted as poor, moderate, good, and excellent reliability, respectively. Kappa values of 0.01-0.20, 0.21-0.40, 0.41-0.60, 0.61-0.80, and 0.81-1 were interpreted as slight, fair, moderate, substantial, and almost perfect agreement, respectively.

## Results

### Demographic and clinical characteristics

A total of 53 cases met the inclusion criteria; after removing repeat fluoroscopic examinations, one examination per dog was analyzed. In 5 dogs, the sternum was outside the fluoroscopic FOV, so these cases were excluded from the diaphragmatic excursion ratio analysis but retained for the other analyses. Clinical severity groups were distributed as follows: no or mild signs of respiratory disease (low-severity group, *n* = 25) and severe signs of respiratory disease (high-severity group, *n* = 28).

Among the 43 dogs with signs of respiratory disease (15/25 from the low-severity group and all 28 from the high-severity group), clinical signs included chronic cough in 38 dogs (88%), respiratory distress in 18 dogs (42%), syncope in 10 dogs (23%), and cyanosis in 3 dogs. Some dogs presented with multiple concurrent signs.

Of the 28 dogs receiving respiratory medication (high-severity group), treatments included bronchodilators in 24 dogs (86%), antitussives in 11 dogs (39%), and corticosteroids or other anti-inflammatory drugs in 5 dogs. Thirteen dogs (46%) received combination therapy with multiple medications.

The study sample consisted of 17 different breeds, with small-breed dogs comprising the majority of cases. The most prevalent breeds were Maltese (*n* = 26, 49%) and Pomeranian (*n* = 10, 19%). Brachycephalic breeds accounted for 17% (*n* = 9) of the total sample, including Shih-Tzu (*n* = 6), French Bulldog (*n* = 1), and Pug (*n* = 2).

The median age was 10 years (IQR, 8-13 years) in the low-severity group and 11 years (IQR, 10-14 years) in the high-severity group, with no statistically significant difference between groups (*P* = .21). The majority of dogs were neutered (castrated males: 27/53, 51%; spayed females: 25/53, 47%), with only one intact female in the low-severity group. Sex distribution did not differ significantly between severity groups (*P* = .41).

### Fluoroscopic findings

In this study, fluoroscopic examination showed various airway and thoracic abnormalities. Mainstem bronchial collapse was present in all dogs and CLLH in 50/53 (94%; 95% CI, 84%-99%). Tracheal kinking occurred in 33/53 (62%; 95% CI, 48%-75%), paradoxical sternal movement in 30/53 (57%; 95% CI, 42%-70%), and cardiac displacement in 22/53 (42%; 95% CI, 28%-56%). Tracheal collapse was observed in all dogs (53/53; 100%). The cervical region predominantly showed mild collapse (52/53; 98%; 95% CI, 90%-100%), while the carina most frequently showed severe collapse (34/53; 64%; 95% CI, 50%-77%). Grade distributions for all regions are shown in Figure 5. The sternal deformity location was typical (caudal) in 44/53 (83%; 95% CI, 70%-92%) and atypical in 9/53 (17%; 95% CI, 8%-30%). The THRR was 0.116 (IQR, 0.091-0.204) overall. The diaphragmatic excursion ratio was measurable in 48 dogs. Reduced excursion (<0.47) was observed in 12/48 (25%; 95% CI, 14%-40%), normal excursion (0.47-0.82) in 18/48 (38%; 95% CI, 24%-53%), and increased excursion (>0.82) in 18/48 (38%; 95% CI, 24%-53%).

### Comparison between clinical severity groups

**Figure 4:**
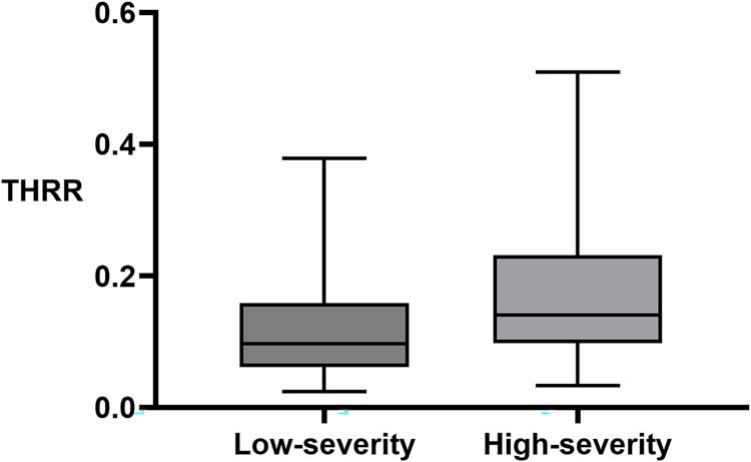
Comparison of THRR between clinical severity groups. Box and whisker plots display the median (horizontal line within box), first and third quartiles (box boundaries), and minimum and maximum values (whiskers). The THRR was significantly higher in the high-severity group [median 0.140 (IQR, 0.100-0.228)] compared with the low-severity group [median 0.097 (IQR, 0.064-0.133)] (*P* = .04). Abbreviation: THRR, thoracic height reduction ratio.

Most fluoroscopic variables did not differ significantly between clinical severity groups (Table 1). However, THRR and paradoxical sternal movement showed statistically significant associations with clinical severity. The THRR was significantly higher in the high-severity group [0.140 (IQR, 0.100-0.228)] compared with the low-severity group [0.097 (IQR, 0.064-0.133)] (*P* = .04; Figure 4). Paradoxical sternal movement was significantly more prevalent in the high-severity group [20/28 (71%; 95% CI, 51%-87%)] than in the low-severity group [10/25 (40%; 95% CI, 21%-61%)] (*P* = .02).

**Table 1:** Proportional distribution of fluoroscopic findings by clinical severity group. Data are presented as count (percentage, 95% CI) unless otherwise stated.

Fluoroscopic feature	Low-severity (*n* = 25)	High-severity (*n* = 28)	*P* value
**PE-intrinsic features**			
**Sternal deformity location**			.72
**Typical**	20/25 (59%-93%)	24/28 (67%-96%)	
**Atypical**	5/25 (59%-93%)	4/28 (59%-93%)	
**Cardiac displacement**	14/25 (35%-76%)	17/28 (41%-78%)	.73
**Concurrent respiratory findings**			
**Mainstem bronchial collapse**	25/25	28/28	-^a^
**Cervical lung lobe herniation**	22/25 (35%-76%)	28/28	.10
**Tracheal kinking**	15/25 (39%-79%)	18/28 (44%-81%)	.75
**Diaphragmatic excursion ratio**			.31
**Reduced (<0.47)**	4/25 (5%-36%)	8/28 (13%-49%)	
**Normal (0.47-0.82)**	11/25 (24%-65%)	7/28 (11%-45%)	
**Increased (>0.82)**	8/25 (15%-54%)	10/28 (19%-56%)	
**Paradoxical sternal movement**	10/25 (21%-61%)	20/28 (51%-87%)	.02

^a^Not applicable (prevalence 100%).

Abbreviation: PE=pectus excavatum.

**Figure 5:**
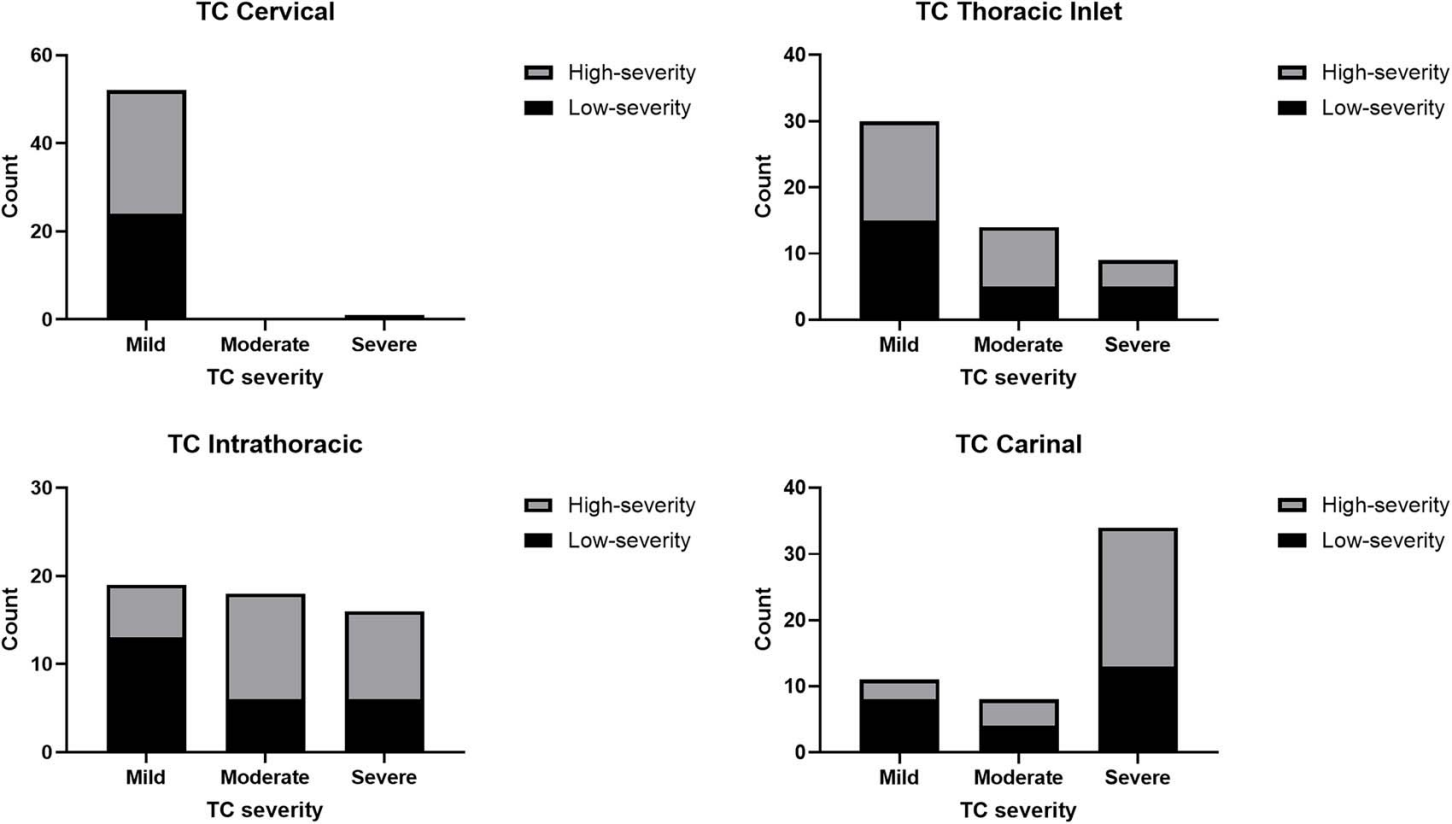
Distribution of tracheal collapse grades across anatomical regions and clinical severity groups. Stacked bar graphs show the proportion of mild (grades 1 and 2), moderate (grade 3), and severe (grade 4) tracheal collapse in 4 anatomical regions (cervical, thoracic inlet, intrathoracic, and carina) between clinical severity groups. No statistically significant differences were observed among groups.

Other fluoroscopic variables, including sternal deformity location (*P* = .72), cardiac displacement (*P* = .73), CLLH (*P* = .10), tracheal kinking (*P* = .75), diaphragmatic excursion ratio categories (*P* = .31), and TC grades at all regions (cervical *P* = .47, thoracic inlet *P* = .65, intrathoracic *P* = .07, and carina *P* = .15; Figure 5), showed no statistically significant associations with clinical severity.

To further evaluate variables as potential independent predictors, univariable logistic regression was performed for variables with *P* < .10 in the above univariable associations (Supplementary Table 1). In multivariable logistic regression, paradoxical sternal movement and intrathoracic TC were included. Thoracic height reduction ratio was excluded due to nonsignificant association and unstable estimates in univariable logistic regression (Supplementary Tables 2 and 3). In the final model, only paradoxical sternal movement remained significantly associated with high clinical severity (odds ratio [OR] 3.71; 95% CI, 1.03-13.4; *P* = .045), whereas intrathoracic TC was not significantly associated (OR 2.87; 95% CI, 0.34-24.3; *P* = .33).

### Interobserver reliability

Interobserver reliability was assessed using ICC for continuous variables (THRR, DER), weighted κ for ordinal variables (TC grades), and Fleiss’ κ for binary variables (cardiac displacement). All reliability measures indicated excellent to almost perfect agreement (ICC/κ > 0.90), demonstrating high consistency among the 3 observers (Table 2). Complete agreement among all observers was achieved for sternal deformity location, BC, CLLH, tracheal kinking, and paradoxical sternal movement.

**Table 2:** Interobserver reliability of fluoroscopic evaluation.

Evaluated feature	Reliability measure	95% CI	Agreement
**Continuous variables (ICC)**			
**THRR**	0.936	0.901-0.962	Excellent
**DER**	0.990	0.981-0.993	Excellent
**Ordinal variables (weighted κ)**			
**TC cervical**	0.919	0.871-0.951	Almost perfect
**TC TI**	0.971	0.954-0.982	Almost perfect
**TC IT**	0.978	0.964-0.986	Almost perfect
**TC CA**	0.956	0.930-0.973	Almost perfect
**Binary variables (Fleiss’ κ)**			
**Cardiac displacement**	0.974	0.819-0.989	Almost perfect

Abbreviations: CA, carina; CE, cervical; DER, diaphragmatic excursion ratio; ICC, intraclass correlation coefficient; IT, intrathoracic; TC, tracheal collapse; THRR, thoracic height reduction ratio; TI, thoracic inlet; κ, kappa.

## Discussion

This study characterized fluoroscopic findings in 53 dogs with PE and examined their associations with clinical severity. Two fluoroscopic variables showed statistically significant associations with clinical severity: THRR was higher in the high-severity group compared with the low-severity group (*P* = .04), and paradoxical sternal movement remained significantly associated with high clinical severity in multivariable analysis (OR 3.71; 95% CI, 1.03-13.4; *P* = .045). However, most other fluoroscopic variables, including cardiac displacement, diaphragmatic excursion ratio, TC grades, and deformity location, showed no statistically significant associations with clinical severity. Notable descriptive findings included BC in all dogs and CLLH in 94% (95% CI, 84%-99%), representing remarkably high prevalence rates. These findings suggest that most fluoroscopic abnormalities represent characteristic features of PE in dogs with limited value, whereas paradoxical sternal movement, particularly when accompanied by greater thoracic height reduction, might serve as a supportive indicator of clinical severity.

Paradoxical sternal movement was observed in 57% (95% CI, 42%-70%) of dogs with PE and showed statistically significant associations with clinical severity. In human medicine, paradoxical sternal movement is recognized as one of the surgical indication criteria and is clinically identified in PE patients, with reduced chest wall motion at the area of the defect during respiration and compensatory increases in abdominal contributions to breathing.^18^^,^^19^ Although the mechanism underlying this phenomenon has not been fully explained in human medicine, it is thought to be associated with chest wall motion dysfunction and limited expansion of the lungs, primarily in the upper lobes.^20^ The high prevalence and association with clinical severity suggest that paradoxical sternal movement might be an important fluoroscopic finding for identifying dogs at higher risk for respiratory compromise.

In our study, the THRR, reflecting the degree of thoracic height reduction at the level of the defect, was significantly higher in the high-severity group compared with the low-severity group. This finding is consistent with human studies demonstrating that greater severity of chest wall deformity is associated with worse cardiopulmonary function, with higher Haller index values observed in patients with pulmonary dysfunction (median 4.7) compared to those without pulmonary disease (median 3.4).^21^

Mainstem bronchial collapse was documented in all dogs in this study, a complete concordance that exceeds the prevalence reported in human patients with PE.^22^ This finding is particularly noteworthy considering that bronchomalacia coexists in 45%-83% of dogs with TC but without PE, and that the overall incidence of bronchomalacia is 50% in dogs undergoing bronchoscopy for respiratory disease.^23^ Mainstem bronchial collapse was frequently observed in all dogs in this study, though comparative studies with PE-free controls are needed to confirm this association.

Human literature reports associations between PE and both dynamic airway collapse and bronchomalacia, with the European Respiratory Society statement listing PE among the causes of tracheomalacia, bronchomalacia, and tracheobronchomalacia.^22^^,^^24^ Mechanical compression through vascular displacement can cause dynamic or static airway collapse and localized folding, specifically with the ascending aorta compressing the lower trachea and right main bronchus, while the pulmonary trunk and left pulmonary artery compress the carina through the left main bronchus.^25^^,^^26^

In addition to mainstem involvement, segmental bronchomalacia of the left main bronchus has been reported in association with PE, and PE might exert an adverse compression effect on tracheobronchomalacia, suggesting that this structural weakness of the airway walls can develop either as a primary condition or secondary to chronic compression.^25^ While the distinction between bronchomalacia and dynamic airway collapse has not been clearly established in dogs,^27^^,^^28^ our study identified bronchial collapse at the mainstem level through fluoroscopy in dogs with PE. These findings indicate that bronchial collapse is frequently observed in dogs with PE, though comparative studies are warranted to confirm the association.

Cervical lung lobe herniation was documented in 94% (50/53) of dogs with PE, substantially higher than the 55.9% prevalence reported in dogs undergoing fluoroscopy for TC screening at the same institution with a similar sample.^16^ Higher intrathoracic pressure is a primary cause of CLLH, with intrathoracic TC contributing to CLLH development through elevated expiratory pressures.^16^ When intrapleural pressure exceeds airway luminal pressure during forced exhalation, dynamic collapse occurs and lung lobes protrude due to elevated pressure. In PE, sternal depression compromises intrathoracic compliance and might amplify respiratory pressure changes.^29^ Combined with the presence of bronchial collapse in all dogs in this study, this altered thoracic mechanics might contribute to CLLH development, suggesting a potential mechanistic relationship.

Our findings contrast with several reported characteristics of PE in dogs. Contrary to earlier reports indicating that PE frequently occurs in brachycephalic dogs,^3^^,^^10^ with 7 of 8 affected dogs documented as brachycephalic breeds, our study found that brachycephalic breeds comprised only 17% (9/53) of cases, with small non-brachycephalic breeds such as Maltese (49%) and Pomeranian (19%) representing the majority. This breed distribution might reflect referral patterns specific to our hospital or geographic region rather than true disease prevalence. Additionally, while a strong positive correlation between clinical severity and atypical PE has been previously reported,^11^ our study found no statistically significant association between deformity location (typical vs atypical) and clinical severity groups (*P* = .72).

Despite the associations observed for THRR and paradoxical sternal movement, most other fluoroscopic variables showed no statistically significant associations with clinical severity. Cardiac displacement was observed in 42% of cases but did not associate with clinical severity (*P* = .73), contrasting with human literature that emphasizes cardiac compression as an indicator of disease severity.^4^^,^^5^ Similarly, the diaphragmatic excursion ratio showed no statistically significant association with clinical severity (*P* = .31), differing from human studies documenting altered diaphragmatic excursion patterns in PE patients.^30^

There are several limitations in this study. As a study conducted at a tertiary referral hospital, selection bias toward cases with greater severity or complications might limit generalizability; however, investigation of referral purposes revealed that many dogs without signs of respiratory disease were included for preoperative evaluation. Clinical severity classification was based on retrospective medical record review over an extended period, and some variability in assessment consistency might have occurred. Furthermore, the classification was treatment-based and therefore might reflect clinical decision-making context (referral indication, clinician preference, and owner factors) as well as true disease severity; associations with severity groups should be interpreted accordingly. Comprehensive respiratory evaluation including bronchoscopy or bronchoalveolar lavage was not performed, and concurrent respiratory diseases could not be ruled out. Functional testing such as pulmonary function tests or echocardiography was not performed, limiting direct assessment of cardiopulmonary impairment. The relatively small sample size (*n* = 53) likely limited our ability to detect small to moderate differences between severity groups, with the study powered to detect only large or large-to-moderate effects. Therefore, the absence of statistically significant associations for certain variables does not exclude or discount clinically relevant effects.

In conclusion, fluoroscopic examination of dogs with PE documented both PE-intrinsic features and concurrent airway abnormalities. Among these findings, paradoxical sternal movement was independently associated with clinical severity, and THRR was higher in the high-severity group. These results indicate that paradoxical sternal movement, particularly when accompanied by greater thoracic height reduction, might serve as a supportive indicator of clinical severity, though comprehensive clinical evaluation remains necessary for accurate severity assessment. Although BC and CLLH did not associate with clinical severity, their remarkably high prevalence suggests an association between PE and these airway abnormalities in dogs, warranting furtherb study.

## Supplementary Material

Supplementary_material_aalag171

## Off-label antimicrobial declaration

The authors declare no off-label use of antimicrobials.

## Institutional animal care and use committee or other approval declaration

The authors declare no institutional animal care and use committee or other approval was needed.

## Abbreviations

BC: mainstem bronchial collapse
CLLH: cervical lung lobe herniation
DER: diaphragmatic excursion ratio
ICC: intraclass correlation coefficient
OR: odds ratio
PE: pectus excavatum
TC: tracheal collapse
THRR: thoracic height reduction ratio
