# Supplementary material for: Fluoroscopic characterization of pectus excavatum in dogs: a cross-sectional study
Source: J Vet Intern Med. 2026 Aug 24;40(4):aalag171. doi: 10.1093/jvimsj/aalag171 (PMC13502407; doi:10.1093/jvimsj/aalag171)
Supplement: Supplementary file 1 [file supplementary_material_aalag171.zip › Video 1 caption.docx]

**Video 1. Paradoxical sternal movement in a dog with pectus excavatum.** Note the sternum moves inward (dorsally) during inspiration and outward (ventrally) during expiration, opposite to normal respiratory mechanics.

**Video 2. Cervical lung lobe herniation in a dog with pectus excavatum.** Note the lung lobe protruding through the thoracic inlet into the cervical region.

**Video 3. Tracheal kinking in a dog with pectus excavatum** **showing abnormal tracheal angulation during respiration.**
