# Supplementary material for: Fluoroscopic characterization of pectus excavatum in dogs: a cross-sectional study
Source: J Vet Intern Med. 2026 Aug 24;40(4):aalag171. doi: 10.1093/jvimsj/aalag171 (PMC13502407; doi:10.1093/jvimsj/aalag171)
Supplement: Supplementary file 1 [file supplementary_material_aalag171.zip › Supplementary tables clean.docx]

**Supplementary Table 1. Univariate analysis**

| **Fluoroscopic feature** | **Odds Ratio** | **95% CI** | ***P* value** |
| --- | --- | --- | --- |
| **THRR** | 88.836 | 0.159-49620.587 | 0.333 |
| **Paradoxical sternal movement** | 4.590 | 1.308-16.104 | 0.017 |
| **TC IT** | 4.333 | 1.093-17.173 | 0.07 |

**Supplementary Table 2. Multivariable logistic regression models with and without THRR**

| **Fluoroscopic feature** | **Model 1 (without THRR) OR (95% CI)** | ***P* value** | **Model 2 (with THRR) OR (95% CI)** | ***P* value** |
| --- | --- | --- | --- | --- |
| **THRR** | – | – | 5.985 (0.007-4982.346) | 0.602 |
| **Paradoxical sternal movement** | 4.590 (1.308-16.104) | 4.590 | 3.997 (1.038-15.384) | 0.044 |

**Supplementary Table 3. Collinearity analysis**

| **Pair** | **Spearman r** | ***P* value** |
| --- | --- | --- |
| **THRR vs Paradoxical** | 0.453 | 0.001 |
